# Utilization of healthcare and prescription medicines after non-pharmacological interventions for depression - A 3-year register follow-up of an RCT in primary care

**DOI:** 10.1016/j.pmedr.2021.101658

**Published:** 2021-12-09

**Authors:** Elisabeth Bondesson, Anna Jöud, Kjerstin Stigmar, Åsa Ringqvist, Martin Kraepelien, Viktor Kaldo, Björn Wettermark, Yvonne Forsell, Ingemar F. Petersson, Maria E.C. Schelin

**Affiliations:** a Lund University, Faculty of Medicine, Department of Clinical Sciences Lund, Division of Orthopaedics, Lund, Sweden; b Skåne University Hospital, Department of Neurosurgery and Pain Rehabilitation, Lund, Sweden; c Lund University, Faculty of Medicine, Department of Laboratory Medicine, Division of Occupational and Environmental Medicine, Lund, Sweden; d Lund University, Division of Physiotherapy, Department of Health Sciences, Lund, Sweden; e Centre for Psychiatry Research, Department of Clinical Neuroscience, Karolinska Institutet, & Stockholm Health Care Services, Region Stockholm, Stockholm, Sweden; f Department of Psychology, Faculty of Health and Life Sciences, Linnaeus University, Växjö, Sweden; g Department of Pharmacy, Faculty of Pharmacy, Uppsala University, Uppsala, Sweden; h Karolinska Institutet, Department of Global Public Health, Stockholm, Sweden; i Department of Research and Education, Skåne University Hospital, Lund, Sweden; j Lund University and Region Skåne, Institute for Palliative Care, Lund, Sweden

**Keywords:** RCT, Randomized control trial, Internet-CBT, Internet-based cognitive behavioural therapy, RR, Risk ratio, ATC, Anatomical Therapeutic Chemical, Depression, Anxiety, Pain, Internet-based treatment, Physical exercise, RCT, Long-term follow-up, Electronic health records, Healthcare utilization, Prescription medicines

## Abstract

Depression is a common, recurrent disorder. There is a need for readily available treatments with few negative side effects, that demands little resources and that are effective both in the short- and long term.

Our aim was to investigate the long-term effectiveness of two different interventions; physical exercise and internet-based cognitive behavioural therapy (internet-CBT), compared to usual care in patients with mild to moderate depression in a Swedish primary care setting.

We performed a register-based 3-year follow-up study of participants in the randomized controlled trial REGASSA (n = 940) using healthcare utilization and dispensed medicines as outcomes.

We found no difference between the three groups regarding *proportion* of participants consulting healthcare due to mental illness or pain during follow-up. Regarding *number* of consultations, there was no difference between the groups, except for consultations related to pain. For this outcome both treatment arms had significantly fewer consultations compared to usual care, during year 2–3, the risk ratio (RR) for physical exercise and internet-CBT was 0.64 (95% CI = 0.43–0.95) and 0.61 (95% CI = 0.41–0.90), respectively.

A significantly lower proportion of patients in both treatment arms were dispensed hypnotics and sedatives year 2–3 compared to the usual care arm, RR for both physical exercise and internet-CBT was 0.72 (95% CI = 0.53–0.98). No other differences between the groups were found.

In conclusion, considering long-term effects, both physical exercise and internet-CBT, being resource-efficient treatments, could be considered as appropriate additions for patients with mild to moderate depression in primary care settings.

Trial registration: The original RCT was registered with the German Clinical Trial Register (DRKS study ID: DRKS00008745).

## Highlights

• A 3-year register follow-up of an RCT for depression interventions was conducted.
• Healthcare utilization and dispensed medicines were used as register outcomes.
• The interventions had no effect on consultations for mental illness during follow-up.
• Both interventions are appropriate additions to mild-moderate depression care.

## 1 Introduction

### 1.1 Background and objectives

Mental illness is a common health problem around the world, major depression is the second most common cause of the global burden of disease (Ferrari et al., 2013). The point prevalence of major depression worldwide has been estimated to 3.6% (Rehm and Shield, 2019) and depression disorders are also highly recurrent (Burcusa and Iacono, 2007). About three-fourths of the individuals affected by depression are managed exclusively in primary care and this disease constitute one of the largest challenges for primary care today (Kroenke and Unutzer, 2017). Depression has a major impact on quality of life for affected individuals and their families, but the disease also has a substantial economic impact on the society. In western countries, depression is the leading cause of sick leave, and further costs are attributable to healthcare and drug utilization (Sandelin et al., 2013). Furthermore, it is well established that patients with depression often have comorbidities, such as anxiety disorders (Lamers et al., 2011, Steffen et al., 2020) and pain disorders (Bair et al., 2003, Bondesson et al., 2018), further adding to their suffering and disability (Arnow et al., 2006, Bair et al., 2003, Bair et al., 2008, DeVeaugh-Geiss et al., 2010).

The most common treatment options for depression today are pharmacological and psychological interventions. Among pharmacological interventions, the most widely used treatment is antidepressants, but also anxiolytics, hypnotics and sedatives are used (The Swedish National Board of Health and Welfare, 2019). However, not all individuals are comfortable using medications, some do not benefit from it and others may experience adverse effects (Cipriani et al., 2018, Sabella, 2018). Different forms of psychotherapy are also available, where cognitive behavioural therapy (CBT) has the most scientific evidence for its effect (Hofmann et al., 2012). In the recent years, CBT has become increasingly available in Swedish primary healthcare. However, given the societal burden of depression, there is still a need for treatments that are readily available, have few negative side effects, demands less resources than the current ones and have positive effects also in the long term.

Internet-based CBT could be one such option, since it has been shown to have treatment effects comparable to face-to-face CBT (Andersson et al., 2019, Păsărelu et al., 2017, Sztein et al., 2018), while allowing the therapist to treat four times as many patients in the same amount of time (Andersson et al., 2014), and is possible to successfully integrate into regular care (Titov et al., 2018). Another promising treatment option is physical exercise, which has been reported to have similar effects to CBT on depression (Hearing et al., 2016). Physical exercise can also be a viable adjunct treatment in combination with usual treatment (Ashdown-Franks et al., 2020, Kvam et al., 2016). Considering the many common comorbidities of depression, it is an advantage that physical exercise has positive effects on these as well, e.g. pain (Geneen et al., 2017) and cardiovascular disease (Gronek et al., 2020).

So far, both internet-CBT and physical exercise have been studied mainly regarding short-term effects on depression while there is a lack of studies investigating the long-term effect (Păsărelu et al., 2017). This would be of particular interest since both treatments aim to provide the patients with tools they can easily implement themselves, without involvement of the healthcare, to maintain positive effects and in the event of a flare or recurrence.

The aim of this study was to investigate the long-term effectiveness of two different interventions; physical exercise and internet-CBT, compared to usual care in adult patients with mild to moderate depression in a Swedish primary care setting.

## 2 Methods

### 2.1 Study design and study sample

The present study is a register-based three-year follow-up study on the randomized controlled trial (RCT), REGASSA, that was conducted between 2011 and 2013 (Hallgren et al., 2015). REGASSA was a multicentre, three-group parallel RCT with the aim to examine the efficacy of two different 12-week interventions for mild to moderate depression disorders. Eligible participants were patients ≥ 18 years of age that scored > 9 on the Patient Health Questionnaire (PHQ-9) (Kroenke et al., 2001) when screened for depression. The study in total included 945 patients. For details on the original RCT see Appendix.

### 2.2 Data sources for the present study

In the present study, we used secondary data from national and regional registers in combination with data collected in the original RCT-study. All data were linked using the Swedish personal identity numbers assigned to all Swedish citizens at birth (Ludvigsson et al., 2009). The dataset was pseudonymized before it was delivered to the research group.

#### 2.2.1 Healthcare consultations

Sweden has universal healthcare i.e. a publicly funded healthcare system with all residents having access to healthcare. The system is decentralised and administered by geographical regions responsible for organizing, delivering and documenting the healthcare. Healthcare data from outpatient specialised care and hospital admissions (including day-care procedures) is stored in the **Swedish National Patient Register** (Ludvigsson et al., 2011). This register has national coverage and is administered by The National Board of Health and Welfare. Primary care data is only stored regionally, and we therefore used both national and regional data sources in this study. We used outpatient specialised care data from the National Patient Register and primary care data from the three (out of the 6 participating) regions contributing the majority of patients in the REGASSA study: i) **Region Stockholm (the Stockholm Regional Health Care Data Warehouse, VAL)**) (Wandell et al., 2013) (n = 610), ii) **Region Skåne (Skåne Healtcare Register, SHR**) (Löfvendahl et al., 2020) (n = 162) and iii) **Region Västra Götaland (VEGA database)** (Osika Friberg et al., 2016) (n = 69). The healthcare databases, both national and regional, contain data from all healthcare consultations, e.g. type of healthcare professional (physician, psychologist, etc), date of consultation and diagnostic codes (classified according to ICD-10), all of which are automatically transferred. From these databases we retrieved personal identification number, diagnosis, sex, date of healthcare consultations and type of healthcare professional the patient consulted.

#### 2.2.2 Dispensed prescription medicines

The **Swedish Prescribed Drug Register** is administered by The National Board of Health and Welfare and includes information on all dispensed prescription medicines from all pharmacies in Sweden (Wallerstedt et al., 2016, Wettermark et al., 2007), from July 2005 and onwards. Medicines in this register are classified according to the Anatomical Therapeutic Chemical (ATC) classification system. From this register we retrieved the following data about the participants’ dispensed medicines: ATC code of each drug, amount dispensed, dosage, and date of dispensing.

#### 2.2.3 Sociodemographic data at baseline

**The Swedish Total Population Register** contains the civil registration of vital events (births, deaths, change of residential address) of the entire Swedish population. Information from this register was used to identify patients who died or migrated.

**The longitudinal integration database for health insurance and labour market studies (LISA)** is held by Statistics Sweden and contains annual demographic data for all residents aged > 15 years (Ludvigsson et al., 2019). From LISA, educational level, civil status, work status, yearly income and information about whether patients were born in Sweden or in another country were collected. For the current study education level was categorised into ‘Low’ (up to 9 years), ‘Medium’ (10–12 years) and ‘High’ (>12 years), married and registered partner formed the category ‘Living together’ while unmarried, divorced and widowed formed the category ‘Living alone’, work status were grouped into ‘Working’ and ‘Not working’ and yearly income level was categorized into tertiles.

#### 2.2.4 Diagnoses at baseline

At baseline, in the original RCT-study, a diagnostic interview, Mini-International Neuropsychiatric Interview (MINI) (Sheehan et al., 1998) was conducted and generated the diagnostic codes.

#### 2.2.5 Self-rated measures at baseline

In the original RCT-study, additional participant information was gathered with a baseline questionnaire, which included body mass index, patients’ affected daily activities, pain and worry (measured by EuroQol EQ-5D)(Rabin and de Charro, 2001), leisure time physical activity level (measured by a 6 level-question), substance use, i.e. hazardous alcohol drinking and daily tobacco use (measured by The Alcohol Use Disorders Identification Test (AUDIT) and by the question: ‘Do you smoke or use tobacco daily?’). The initial six levels of physical activity were categorized into: 1) inactive, 2) light physical activity and 3) moderate to vigorous exercise.

### 2.3 Outcomes

#### 2.3.1 Healthcare utilization

We studied the proportion of participants having any physical healthcare consultation in ambulatory care (both primary care and outpatient specialised care), as well as the total number of consultations for mental illness or pain. A consultation for mental illness was defined as having a registered ICD-10 code of ‘F’ (chapter Mental, Behavioural and Neurodevelopmental disorders) including all subcategories or consulting a psychologist, psychiatrist or counsellor. A consultation for pain was defined as an ICD-10 code of abdominal pain, headache, back/neck pain, joint pain/myalgia, pain not specified in primary care, or persistent pain being registered (ICD-10 codes ‘R10′, ‘G43′, ‘G44′, ‘R51′, ‘M54′, ‘M25.5′, ‘M79.1′, ‘M79.6′ ‘R52-‘, ‘R52.9′, ‘M79.7′, ‘R52.1′, ‘R52.2′ or ‘F45.4′ including all subcategories). We present the result for year 1–2 and 2–3 after inclusion in the original RCT, throughout.

#### 2.3.2 Dispensed prescription medicines

We further investigated the proportion of participants being dispensed at least one prescription of antidepressants (ATC-N06A), anxiolytics (ATC-N05B), hypnotics and sedatives (ATC-N05C) and/or opioids (ATC-N02A) as well as the number of dispensed defined daily doses (DDDs) of these drugs. As with healthcare consultations, we present the result per year 1–2 and 2–3 after inclusion in the original RCT.

### 2.4 Statistical methods

Differences in baseline characteristics were assessed using independent samples t-tests for continuous data and chi-squared tests for categorical data. Regarding the effect of the interventions, the comparison was based on the intention-to-treat principle, where the participants are analysed according to the group they were randomized to in the original RCT-study, regardless of their degree of adherence. As our main interest was the overall effect of the physical exercise intervention, the three intensity groups were analyzed together.

Data on dispensed daily doses of medication were transformed to count data by rounding summed daily doses per individual to nearest integer. For both healthcare consultations and dispensed daily doses we converted extreme values, defined as values over the 99 percentile, to the 99 percentile value. Since the outcome variables had a zero-inflated and over-dispersed distribution, the comparison between groups, internet-CBT, physical exercise and usual care, were analysed in a two-step manner. In step one we used modified Poisson regression (i.e. Poisson regression with a robust error variance, using the proc genmod procedure and the repeated subject statement in SAS) (Zou, 2004) to assess whether the participants had a) any healthcare consultation for mental illness/pain or not and b) any dispensed prescription of antidepressants/anxiolytics/hypnotics and sedatives/opioids or not, i.e. a binary outcome. In step two we used Pearson-scale-adjusted negative binomial regression (using the proc genmod procedure in SAS) to model the count variables: a) mean number of healthcare consultations among those with any consultation and b) mean number of dispensed daily doses of antidepressants, anxiolytics, hypnotics and sedatives or opioids among those with any prescription, i.e. patients with no consultations/prescriptions were excluded in step two.

All statistical analyses were conducted using the SAS software, version Enterprise 8.1. (SAS Institute Inc.).

The original study was approved by the Regional Ethical Review Board in Stockholm, Sweden (Registration number 2010/1779-31/4). All patients provided written informed consent. For this new phase, an ethical approval was sought and approved and included an opt-out option for the original participants (Regional Ethical Review Board in Stockholm, Sweden, Dnr 2015/2112-31).

## 3 Results

**Fig. 1:**
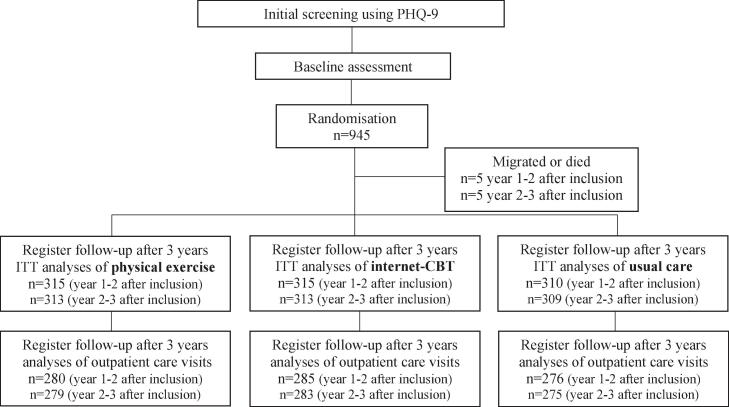
Flowchart of number of patients included in the different analyses.

In the original RCT study 945 patients were included. During the three-year follow-up period, 10 patients died or migrated, leaving 940 patients for analyses year 1–2 after inclusion and 935 patients for year 2–3. For healthcare consultations, we had data from 841 participants (89%) year 1–2 and 837 (90%) year 2–3 (Fig. 1).

Most participants were female (73%), the sample’s mean age was 43 years (s.d. = 12) and most had a combined depressive and anxiety disorder (77%). Many patients had a higher education, and the majority was working (80%). At baseline, a significant proportion reported physical pain (68%) and the physical activity level was mainly light. A total of 16% reported heavy use of alcohol and 21% used tobacco (Table 1). There was no statically significant difference between the three intervention groups at baseline regarding any of the socioeconomic or clinical characteristics and therefore we present the study population as a whole (Table 1).

**Table 1:** Sample characteristics.

Characteristics	All participants N = 940
*Sociodemographic data and diagnosis*	
Female sex, n (%)	686 (73)
Age at inclusion, mean (SD)	43 (12)
Country of birth, n (%) Born in Sweden	755 (80)
MINI^a^ diagnoses, n (%) Mood disorder (only) Anxiety disorder (only) Both mood and anxiety disorder	84 (9) 126 (14) 701 (77)
Education level^b^, n (%) Low Medium High	44 (5) 382 (41) 508 (54)
Civil status, n (%) Living together Living alone	298 (32) 639 (68)
Work status, n (%) Working Not working	748 (80) 189 (20)
Yearly income (EUR)^c^ n (%) National lower tertile National middle tertile National higher tertile	332 (35) 338 (36) 270 (29)
*Self-rated measures*	
BMI mean (SD)	26 (5)
Health status^d^, n (%) Daily activities affected^e^ Moderate or severe pain^f^ Highly worried^g^	373 (40) 636 (68) 363 (39)
Physical activity level, n(%) Inactive Light Moderate to vigorous	141 (15) 457 (49) 339 (36)
Substance use, n (%) Hazardous drinking^h^ Daily tobacco use	153 (16) 193 (21)

a MINI, Mini-international Neuropsychiatric Interview.

b Education level: low (up to 9 years), medium (10–12 years), high (>12 years).

c Yearly income in tertiles: lower (up to 16 104 EUR), middle (16 104-23 734 EUR) Higher (>23 734 EUR) (1 EUR 1 = 1,103 USD, FOREX 2020-02-05).

d Health status from EQ-5D.

e Answer options 2 and 3 presented together.

f Answer options 2 and 3 presented together.

g Answer option 3 presented.

h The recommended (AUDIT) cut-off scores used to identify hazardous drinkers were > 8 for men and 6 for women (Strid et al., 2019). Missing, variable (n): MINI diagnoses (29) Education level (6), Civil status (3), Work status (3), Bmi (14), Health status (1, 6, 2), Physical activity level (3), Substance use (5, 2).

### 3.1 Healthcare utilization

On average 43% of the patients in the study had one or more consultation annually for mental illness during the follow-up period. Among those with any consultation for mental illness the average number of consultations was 5 per year. The majority (76%) of healthcare consultations for mental illness were in primary care. There was no difference between the groups, neither regarding *proportion* of participants with any healthcare consultation for mental illness, nor regarding the *number* of healthcare consultations, during any of the follow-up periods (Table 2).

**Table 2:** Healthcare utilization per intervention group.

*Proportion of participants with visits to outpatient care for **mental illness**, 1–2 years and 2–3 years after inclusion, per intervention group*					
Mental illness	Proportions			RR^a^ (95% CI)	
	PE	ICBT	UC	PE vs UC	ICBT vs UC
Outpatient care (%)					
Year 1–2	46	44	47	0.98 (0.82–1.17)	0.93 (0.78–1.12)
Year 2–3	40	37	42	0.95 (0.78–1.16)	0.89 (0.72–1.09)

*Number of healthcare visits to outpatient care among those with one or more healthcare visits for **mental illness**, 1-2 years and 2-3 years after inclusion, per intervention group*					
Mental illness	Mean number			RR^b^ (95% CI)	
	PE	ICBT	UC	PE vs UC	ICBT vs UC

Outpatient care					
Year 1–2	4.63	4.85	4.94	0.92 (0.71–1.18)	1.00 (0.78–1.29)
Year 2–3	4.54	5.66	5.74	0.77 (0.59–1.02)	1.02 (0.77–1.34)

*Proportion of participants with visits to outpatient care for **pain**, 1-2 years and 2-3 years after inclusion, per intervention group*					
Pain	Proportions			RR^a^ (95% CI)	
	PE	ICBT	UC	PE vs UC	ICBT vs UC

Outpatient care (%)					
Year 1–2	31	33	34	0.90 (0.71–1.15)	0.97 (0.77–1.22)
Year 2–3	34	33	33	1.04 (0.82–1.32)	1.00 (0.79–1.27)

*Number of healthcare visits to outpatient care among those with one or more healthcare visits for **pain**, 1-2 years and 2-3 years after inclusion, per intervention group*					
Pain	Mean number			RR^b^ (95% CI)	
	PE	ICBT	UC	PE vs UC	ICBT vs UC

Outpatient care					
Year 1–2	2.99	3.04	3.60	0.72 (0.46–1.13)	0.72 (0.46–1.12)
Year 2–3	3.07	3.05	4.24	***0.64 (0.43***–***0.95)***	***0.61 (0.41***–***0.90)***

PE physical exercise, ICBT Internet-based cognitive behavioural therapy, UC Usual care.

RR^a^ relative risk (Modified Poisson regression), CI confidence interval, RR^b^ relative risk (Negative binomial regression).

During each year, one third of all patients, 33%, had consultations related to any pain diagnosis and among those the average number of consultations was 3 per year. Most healthcare consultations related to pain were in primary care, 89%. We found no difference between groups regarding *proportion* of participants consulting healthcare for pain. Regarding *number* of healthcare consultations, we found less consultations for both treatment arms compared to usual care during year 2–3. The physical exercise group had an RR of 0.64 (95% CI: 0.43–0.95) compared to usual care. For the internet-CBT group the RR was 0.61 (95% CI: 0.41–0.90) (Table 2).

### 3.2 Dispensed prescription medicines

On average, 36% of all patients were dispensed antidepressants each year, the corresponding proportion for anxiolytics was 16% and for hypnotics and sedatives it was 19%. We found no difference between the groups regarding *proportion* of participants being dispensed prescription medicines, neither antidepressants nor anxiolytics. For hypnotics and sedatives however, a lower proportion were dispensed these medications year 2–3 after inclusion for both treatment arms as compared to usual care. Both the physical exercise group and the internet-CBT group had 28% lower risk of being dispensed a prescription compared to the usual care group, RR was 0.72 (95% CI: 0.53–0.98) for both groups (Table 3).

**Table 3:** Dispensed prescription medicines per intervention group.

*Proportion of participants with prescription of antidepressants, anxiolytics, hypnotics and sedatives, 1–2 years and 2–3 years after inclusion, per intervention group*					
Psychotropics	Proportions			RR^a^ (95% CI)	
	PE	ICBT	UC	PE vs UC	ICBT vs UC
Antidepressants (%)					
Year 1–2	35	39	37	0.94 (0.76–1.16)	1.06 (0.87–1.30)
Year 2–3	34	37	36	0.95 (0.77–1.18)	1.03 (0.84–1.27)
Anxiolytics (%)					
Year 1–2	17	15	15	1.20 (0.84–1.73)	1.05 (0.72–1.53)
Year 2–3	18	17	15	1.16 (0.81–1.65)	1.09 (0.76–1.57)
Hypnotics and sedatives (%)					
Year 1–2	17	17	17	0.95 (0.67–1.34)	1.00 (0.71–1.41)
Year 2–3	18	18	25	***0.72 (0.53***–***0.98)***	***0.72 (0.53***–***0.98)***

*Dispensed volumes (DDDs) of antidepressants, anxiolytics, hypnotics and sedatives among those with medication, 1–2 years and 2–3 years after inclusion, per intervention group*					

Psychotropics	Mean number			RR^b^ (95% CI)	
	PE	ICBT	UC	PE vs UC	ICBT vs UC

Antidepressants					
Year 1–2	384	346	330	1.16 (0.96–1.41)	1.05 (0.87–1.26)
Year 2–3	352	378	331	1.06 (0.88–1.28)	1.14 (0.95–1.37)
Anxiolytics					
Year 1–2	62	34	43	1.43 (0.85–2.39)	0.79 (0.46–1.35)
Year 2-3^c^	53	41	51	1.03 (0.61–1.75)	0.81 (0.47–1.38)
Hypnotics and sedatives					
Year 1–2	194	171	157	1.23 (0.82–1.86)	1.09 (0.73–1.63)
Year 2–3	151	169	129	1.18 (0.80–1.73)	1.31 (0.89–1.93)

*Proportion of participants with prescription of Opioids, 1–2 years and 2–3 years after inclusion, per intervention group*					

Opioids	Proportions			RR^a^ (95% CI)	
	PE	ICBT	UC	PE vs UC	ICBT vs UC

Opioids (%)					
Year 1–2	11	11	12	0.88 (0.57–1.36)	0.88 (0.57–1.36)
Year 2–3	11	12	13	0.84 (0.55–1.29)	0.87 (0.57–1.32)

*Dispensed volumes (DDDs) of opioids among those with medication, 1–2 years and 2–3 years after inclusion, per intervention group*					

Opioids	Mean number			RR^b^ (95% CI)	
	PE	ICBT	UC	PE vs UC	ICBT vs UC

Opioids					
Year 1–2 ^d^	33	38	42	0.79 (0.46–1.34)	0.90 (0.53–1.53)
Year 2–3 ^e^	32	34	49	0.66 (0.36–1.22)	0.70 (0.38–1.28)

PE physical exercise, ICBT Internet-based cognitive behavioural therapy, UC Usual care.

Antidepressants = ATC-N06A, Anxiolytics = ATC-N05B, Hypnotics and sedatives = ATC-N05C, Opioids = N02A.

RR^a^ relative risk (Modified Poisson regression), CI confidence interval, RR^b^ relative risk, (Negative binomial regression), DDD = Defined Daily Dose.

^c^ dispensed daily doses missing = 1, ^d^ dispensed daily doses missing = 1, ^e^ dispensed daily doses missing = 3.

The *number* of dispensed daily doses among those with dispensed prescriptions did not differ between the groups for any of the investigated psychotropic medicines. Patients had on average 356 dispensed daily doses of antidepressants each year, the corresponding number for anxiolytics was 47 and for hypnotics and sedatives it was 162 (Table 3).

On average 12% of the patients were dispensed at least one prescription of opioids each year and for those the average dispensed daily dose during a year was 38. No difference was found between the groups, neither regarding *proportion* of participants who were dispensed opioids nor regarding *number* of dispensed daily doses (Table 3).

## 4 Discussion

In this study we used national and regional health registers to assess the long-term effects after an original RCT designed to study the impact of physical exercise and internet-CBT on depression. The proportion of patients who consulted healthcare or used prescription medicines were largely the same across the different groups during the follow-up period. Also, the number of healthcare consultations and dispensed medicines were similar. The exceptions were consultations for pain and dispensed prescriptions of hypnotics and sedatives, which were less in both treatment arms compared to usual care. Thus, though the effect is limited, both physical exercise and internet-CBT may be valuable as potential treatment additions. Anxiety has earlier been reported as a common comorbidity among patients with depression (Lamers et al., 2011, Steffen et al., 2020). In this study we found that most patients (77%) fulfilled diagnostic criteria for both a depressive and an anxiety disorder. The results of this study must be interpreted in the light of this. However, both internet-CBT and physical exercise have previously been reported to be effective for anxiety disorders as well as depression (Andersson et al., 2019, Ashdown-Franks et al., 2020, Aylett et al., 2018, Olthuis et al., 2016, Păsărelu et al., 2017).

It is well known that primary care utilization in patients with depression is high (Donohue and Pincus, 2007), but few studies have investigated the effectiveness of internet-CBT or physical exercise on healthcare utilization. In a study conducted in the US, a decline in “high healthcare utilization” was reported after 6 months for patients in primary care who had received treatment for anxiety with a digital cognitive behavioural program, compared to a matched group who received ordinary treatment (Oser et al., 2019). In the US study “High utilization” was defined as ≥ 4 outpatient medical consultations over a 6-month period (Oser et al., 2019). Our results point in the same direction, but only concerning consultations for pain 2–3 years after inclusion. In a Swedish RCT of primary-care patients diagnosed with depression, healthcare consultations were compared between two groups (internet-CBT and usual care) after 3 months. There were, as expected, significantly more therapist contacts in the intervention group, but there was no difference in the number of consultations to GPs or nurses, or in number of telephone contacts (Eriksson et al., 2017). Eriksson et al. also followed use of medication for up to one year and found that the use of antidepressants and sedatives did not differ between treatment groups at the 6- and 12-month follow-ups (Eriksson et al., 2017). This result is also in line with our findings, where, in addition to antidepressants also anxiolytics and opioids were prescribed to patients in all three groups to the same extent up to three years after inclusion in the study. However, in our study hypnotics and sedatives were prescribed slightly less during year 2–3 in both treatment arms compared to usual care.

Since our results indicate that internet-CBT and physical exercise are equally, or only slightly more effective than usual care, it could be argued that they do not contribute to the treatment strategy for this patient group in the long run. However, as previously reported, change in the primary outcome (depressive symptoms) was significantly superior for these two interventions compared to usual care, both directly after treatment and at the one year follow-up (Hallgren et al., 2016, Hallgren et al., 2015). In addition, we would like to direct attention to some important advantages of both internet-CBT and physical exercise, essential for handling these patients in primary care. Both treatments are readily available and have few negative side effects. Internet-CBT has a modest cost to the patient as Sweden has a universal publicly funded healthcare system with all residents having access to healthcare at reasonable expenditure levels. Many employers also offer their employees benefits such as subsidized gym classes/memberships and/or the possibility to work out on paid time, as this is tax free. Moreover, internet-CBT allows the patient to themselves choose the time to perform the treatment without having to come to the clinic for e.g. weekly sessions. This could be an advantage for patients who live in rural communities and also for those who wish to remain at work or in studies while simultaneously be in treatment. In the original REGASSA study the physical exercise intervention had a structured approach. Participants exercised together with others in groups and for those that missed out on sessions personal reminders were sent. This approach could have influenced the effect of the intervention. However, physical exercise is a feasible intervention that can be performed upon the individuals own preference of time and place. In addition, physical exercise has many other positive health effects relevant for the comorbidities common in mental illness (Bair et al., 2008, Cohen et al., 2015, Roy and Lloyd, 2012). Another important aspect is that the internet-CBT and physical exercise are likely to be cost-effective compared to usual care according to a recent published study (Kraepelien et al., 2018).

The main strength of the present study is the combination of a randomized controlled design with a register-based follow-up. While the RCT design allows control for both measured and unmeasured confounding, the registry-based study enabled cost-effective long-term follow-up data of effectiveness in a real-life primary care setting, thus increasing generalisability. One limitation, though, is that it is unknown to us how many individuals that were offered to participate in the original trial and thus how many declined. If those who did participate in the study were systematically different from those who declined, e.g. in baseline severity of the depression, this could potentially limit generalisability of the results from the study. Since our study population consisted of working-age individuals, children, adolescents and older age groups are less represented, as is to be expected. Although we do not have reasons to believe that our results would differ, we cannot be certain the result holds for the youngest and oldest age groups of the population. Our sex distribution, with most of the study participants being women, is representable among those diagnosed with depression in the total population of Sweden (Wallerblad et al., 2012). Another limitation is the low compliance rate to the interventions in the original RCT, which can lead to misclassification and complicate the interpretation of the results. Compliance has been a challenge in other studies as well, Oser et al reported that only 52% of the patients who were invited to participate downloaded the studied internet-CBT treatment app and only 38% started the program (Oser et al., 2019). Higher participation rates, around 70%, were reported in a study of three different internet-CBT programs in primary care in Australia (Newby et al., 2017). They also conducted post-hoc analyses to explore factors that contributed to the differences in course completion rates, their results suggested that the completers had less severe psychological distress and were older (Newby et al., 2017). In our study population, the overall compliance to the treatment predicted reductions in depression within the internet-CBT arm (Kraepelien et al., 2019), suggesting a dose–response relationship. Methods to increase compliance need to be further explored and in a recently published study protocol this is one of the aims (Bell et al., 2020). Further, and relating to compliance, we have no information on whether or to what extent participants continued to use the strategies they learned during their intervention sessions, for example if they continued to exercise or to use new behaviors during the follow-up period. Finally, we used register data to assess the effect of the treatments which to some extent can limit the level of detail obtained, as compared to self-reports. However, we believe that the benefits of objectiveness of the data and completeness of follow-up more than compensates for this. It also enabled a longer follow-up period than commonly used in RCTs.

A significant proportion of patients (68%) in the current study reported physical pain, highlighting the comorbidity between mental illness and pain shown in earlier studies (Bair et al., 2003, Bair et al., 2008, Bondesson et al., 2018). This made it important to also investigate healthcare consultations for pain and pain medication. From a clinical point of view, it is interesting that the number of healthcare consultations for pain was lower, both among patients in the physical exercise- and internet-CBT treatment arms, compared to the usual care group. In the case of medications for pain some analgesics, e.g. paracetamol and anti-inflammatory drugs may be purchased over the counter at pharmacies. Consequently, we decided to restrict our analyses to opioids. Furthermore, the use of opioids, especially among patients with chronic pain, is a health concern in many countries (Meyer et al., 2020).

It cannot be excluded that the investigated interventions could have *trans*-diagnostic effects. Other studies have already argued that this is the case both for physical exercise and internet-CBT when treating different mental disorders (Ashdown-Franks et al., 2020, Titov et al., 2015). Both interventions focus on providing increased coping skills to patients, which is important for managing both mental illness and pain disorders.

One important implication for future research is the need to gain knowledge on how to increase attendance and compliance to treatment which, as previously discussed, is low in many studies, including the present one. It is obviously not enough that treatments are effective if patient adherence is lacking, and the way to achieve this should be through thorough research, e.g. randomizing the strategy for reminders/encouragement to participate.

In conclusion, differences between treatment groups regarding healthcare consultations and dispensed prescription medicines were very small but in favour of the treatment arms. Given these results, both physical exercise and internet-CBT, being resource-efficient treatments, could be considered as relevant additions for patients with mild to moderate depression in primary care settings and could possibly lead to improved long-term self-management of sleep, anxiety, and pain.

## 5 Funding sources

This study was supported by the Swedish Research Council for Health, Working life, and Welfare (FORTE, grant no. 2014-02180).

### CRediT authorship contribution statement

**Elisabeth Bondesson:** Conceptualization, Methodology, Data curation, Writing – original draft, Formal analysis, Writing – review & editing. **Anna Jöud:** Conceptualization, Methodology, Writing – original draft, Formal analysis, Writing – review & editing, Resources, Funding acquisition. **Kjerstin Stigmar:** Writing – original draft, Writing – review & editing. **Åsa Ringqvist:** Writing – review & editing. **Martin Kraepelien:** Writing – review & editing. **Viktor Kaldo:** Writing – review & editing. **Björn Wettermark:** Writing – review & editing. **Yvonne Forsell:** Writing – review & editing. **Ingemar F. Petersson:** Writing – review & editing, Funding acquisition. **Maria E.C. Schelin:** Conceptualization, Methodology, Writing – original draft, Formal analysis, Writing – review & editing, Funding acquisition.

## Declaration of Competing Interest

The authors declare the following financial interests/personal relationships which may be considered as potential competing interests: Åsa Ringqvist declares: Lectures on chronic pain for medical professions arranged and paid for by Sanofi. No recommendations on medication is provided during or following lectures. All other authors declare no conflicts of interest.
