# Supplementary material for: Utilization of healthcare and prescription medicines after non-pharmacological interventions for depression - A 3-year register follow-up of an RCT in primary care
Source: Prev Med Rep. 2021 Dec 9;25:101658. doi: 10.1016/j.pmedr.2021.101658 (PMC8800057; doi:10.1016/j.pmedr.2021.101658)
Supplement: Supplementary data 1 [file mmc1.docx]

**Appendix**

The original REGASSA study was a multicentre, three-group parallel RCT carried out in twenty primary care units in six different healthcare regions in Sweden. The target group was the adult working population. The aim of the original study was to examine the efficacy of two different 12-week interventions for mild to moderate depression disorders and the main outcome was change in depression severity. Recruitment began in February 2011 and the last participants finished treatment in March 2013. Patients were partly recruited at the different primary healthcare units and partly by advertising in newspapers. Patients ≥18 years of age were screened for depression using the Patient Health Questionnaire (PHQ-9) (Kroenke et al., 2001) and those who scored >9 on PHQ-9 were invited to participate in the trial. Exclusion criteria were: severe somatic illness, a primary alcohol- or drug use disorder or a psychiatric diagnosis that required specialist treatment (such as psychosis). All included patients (n=945) were randomized to either physical exercise (n=316), internet-CBT (n=317) or to usual care by a physician in primary healthcare (n=312). Assessment of depression severity was conducted at baseline (pre-randomization), directly after the intervention (at 3 months) and at 12-months. A detailed description of the study design, interim findings and results at 12 months has previously been reported (Hallgren et al., 2016; Hallgren et al., 2015). These publications have established superior effects of internet-CBT and physical exercise compared with usual care for depression (Hallgren et al., 2016; Hallgren et al., 2015) as well as sleep, stress and psychological functioning (Strid et al., 2016). Different exercise intensity rendered no or rather small differences regarding change in depression score (Helgadóttir et al., 2017; Helgadóttir et al., 2016). However, all three interventions were equally effective in reducing sick leave and unemployment after 12 months (Kaldo et al., 2018).

There were no restrictions in the original RCT concerning ongoing care, patients could e.g. continue to use any prescribed pharmacological treatment or receive a new prescription of medicine. The interventions internet-CBT and physical exercise were thus offered in addition to usual care and was free of charge.

*Internet-based CBT*

Patients randomized to internet-CBT (n=317) received treatment through a secure website operated through the Stockholm county council healthcare services. Online forms were completed to identify patient-specific mental health concerns and work-related problems.

Patients worked through a self-help manual in the form of modules. During the first few weeks the modules addressed problems related to depressive symptoms in general, for instance inactivity and avoidance behaviours. Later in the treatment process, modules were individually tailored to patient-specific problems such as different comorbid symptoms often seen in depression (worry, panic attacks, social anxiety, stress, insomnia and pain). Other patient-specific modules focused on work-related problems and motivated the patient to return to work. The manual was mostly text-based, but also included images and sound clips (Kraepelien et al., 2019).

Patient responses were monitored by the assigned clinician on a weekly basis and high-risk individuals received additional help if necessary. Adherence and perceived progress in therapy was monitored through digital homework reports and patients could only move on to the next module if the previous module had been completed and the patient had received feedback from their assigned psychologist. Inactive participants were contacted by their assigned psychologist and encouraged to continue. On average, the psychologist spent 16 min per participant per treatment week (Kraepelien et al., 2018) and patients accessed eight internet-CBT modules out of an expected 13 (adherence rate 60%) (Hallgren et al., 2016). The internet-CBT documents were available an additional 6 months after the intervention was terminated. However, patients could no longer communicate with the psychologists during this period.

*Physical exercise*

Patients in the exercise group in the original RCT (n=316) were further randomized to one of three intensity groups: light exercise such as yoga or stretching classes, moderate exercise such as low-intermediate aerobics classes, and vigorous exercise which consisted of middle-intensity aerobics. All classes were supervised and took place at “Friskis och Svettis”, a modern fitness centre service with locations throughout Sweden, and were managed by qualified trainers. Patients were requested to complete three one-hour sessions per week for 12 weeks. Adherence to the exercise regimen was monitored through weekly face-to-face meetings with a trainer or physiotherapist. Participants who did not attend any scheduled sessions for 1 week or more were contacted by telephone. Also, text-messages were used to encourage continuation of exercises. Participants attended an average of 14.5 one-hour classes out of 36 possible, which corresponds to approximately 40% of the recommended classes (Strid et al., 2019). Patients were also on avarage present at 4,9 weekly physiotherapist sessions out of 12 and received on average 7.1 phone reminders to participate in class (Kraepelien et al., 2018). The period with exercise free of charge ended along with the termination of the intervention, i.e. 12 weeks after inclusion. However, it was possible to continue at regular classes at the same fitness centre at one’s own expense.

*Usual care*

Participants randomized to usual care in the original RCT (n=312) received standard treatment for depression as decided by their general medical practitioner (GP). During the 12-week treatment period patients received, on average, between one and two consultations to a GP, which was similar to the number of GP consultations for the patients randomized to internet-CBT and physical exercise. Further, in the usual care group 20% had counselling, 9% had individual CBT and 8% had other psychological treatments, corresponding figures for the internet-CBT group were 6%, 3% and 5% and for the physical exercise group 13%, 3% and 6% (Kraepelien et al., 2018). Almost one-fourth of patients in the usual care group reported receiving no treatment at all (Strid et al., 2019).

**Author contributions in original RCT study**
Martin Kraepelien: Conceptualization, design, data collection/curation.
Viktor Kaldo: Conceptualization, design, data collection/curation.
Yvonne Forsell: Conceptualization, design, data collection/curation.

**References**

Hallgren, M., Helgadóttir, B., Herring, M.P., Zeebari, Z., Lindefors, N., Kaldo, V., Öjehagen, A., Forsell, Y., 2016. Exercise and internet-based cognitive-behavioural therapy for depression: multicentre randomised controlled trial with 12-month follow-up. The British journal of psychiatry : the journal of mental science 209:414-20. doi: 10.1192/bjp.bp.115.177576

Hallgren, M., Kraepelien, M., Ojehagen, A., Lindefors, N., Zeebari, Z., Kaldo, V., Forsell, Y., 2015. Physical exercise and internet-based cognitive-behavioural therapy in the treatment of depression: randomised controlled trial. The British journal of psychiatry : the journal of mental science 207:227-34. doi: 10.1192/bjp.bp.114.160101

Helgadóttir, B., Forsell, Y., Hallgren, M., Möller, J., Ekblom, Ö., 2017. Long-term effects of exercise at different intensity levels on depression: A randomized controlled trial. Preventive medicine 105:37-46. doi: 10.1016/j.ypmed.2017.08.008

Helgadóttir, B., Hallgren, M., Ekblom, Ö., Forsell, Y., 2016. Training fast or slow? Exercise for depression: A randomized controlled trial. Preventive medicine 91:123-31. doi: 10.1016/j.ypmed.2016.08.011

Kaldo, V., Lundin, A., Hallgren, M., Kraepelien, M., Strid, C., Ekblom, Ö., Lavebratt, C., Lindefors, N., Öjehagen, A., et al., 2018. Effects of internet-based cognitive behavioural therapy and physical exercise on sick leave and employment in primary care patients with depression: two subgroup analyses. Occupational and environmental medicine 75:52-58. doi: 10.1136/oemed-2017-104326

Kraepelien, M., Blom, K., Lindefors, N., Johansson, R., Kaldo, V., 2019. The effects of component-specific treatment compliance in individually tailored internet-based treatment. Clinical psychology & psychotherapy 26:298-308. doi: 10.1002/cpp.2351

Kraepelien, M., Mattsson, S., Hedman-Lagerlöf, E., Petersson, I.F., Forsell, Y., Lindefors, N., Kaldo, V., 2018. Cost-effectiveness of internet-based cognitive-behavioural therapy and physical exercise for depression. BJPsych open 4:265-73. doi: 10.1192/bjo.2018.38

Kroenke, K., Spitzer, R.L., Williams, J.B., 2001. The PHQ-9: validity of a brief depression severity measure. Journal of general internal medicine 16:606-13. doi: 10.1046/j.1525-1497.2001.016009606.x

Strid, C., Andersson, C., Forsell, Y., Öjehagen, A., Lundh, L.G., 2016. Internet-based cognitive behaviour therapy and physical exercise - Effects studied by automated telephone assessments in mental ill-health patients; a randomized controlled trial. The British journal of clinical psychology 55:414-28. doi: 10.1111/bjc.12111

Strid, C., Hallgren, M., Forsell, Y., Kraepelien, M., Öjehagen, A., 2019. Changes in alcohol consumption after treatment for depression: a secondary analysis of the Swedish randomised controlled study REGASSA. BMJ open 9:e028236. doi: 10.1136/bmjopen-2018-028236
